# Episodic Ataxia Associated With Synaptosomal-Associated Protein 25 (SNAP25) Variant: Beyond Epilepsy and Developmental Delay

**DOI:** 10.7759/cureus.102586

**Published:** 2026-01-29

**Authors:** Inês F Fernandes, Ana Cristina Figueiredo, João Parente Freixo, Juliette Dupont, João Carvalho

**Affiliations:** 1 Pediatrics, São Bernardo Hospital, Arrábida Local Health Unit, Setúbal, PRT; 2 Pediatric Neurology, Torrado da Silva Child Development Center, Garcia de Orta Hospital, Almada–Seixal Local Health Unit, Almada, PRT; 3 Genetics, Center for Predictive and Preventive Genetics, Institute of Molecular and Cell Biology, University of Porto, Porto, PRT; 4 Genetics Service, Santa Maria Local Health Unit, Lisbon Academic Medical Center, Lisbon, PRT

**Keywords:** ataxia, case report, developmental and epileptic encephalopathies, intellectual impairment, seizures, snap25

## Abstract

Developmental and epileptic encephalopathies (DEEs) of infancy and childhood are characterized by early-onset seizures and developmental impairment. Heterozygous missense or loss-of-function variants in synaptosomal-associated protein 25 (SNAP25), a core component of the presynaptic soluble N-ethylmaleimide-sensitive factor attachment protein receptor (SNARE) complex, have been implicated in a spectrum of DEEs with variable neurological features. We report a male child carrying a novel de novo splice-site variant in SNAP25, expanding the known mutational and phenotypic spectrum of this condition.

The patient presented at 32 months with afebrile seizures since seven months of age, with frequent seizure clusters and status epilepticus. He exhibited a moderate global developmental delay and recurrent, transient episodes of gait ataxia triggered by febrile illnesses, lasting up to one week and resolving without residual deficits. Neurological examination revealed mild microcephaly and a clumsy gait without persistent ataxia or weakness between febrile episodes. Brain magnetic resonance imaging (MRI) was normal, and the electroencephalogram (EEG) showed bilateral frontal paroxysmal activity. Genetic testing identified a heterozygous SNAP25 variant (NM_003081.5:c.114+2dup), predicted to abolish the donor splice site and confirmed as de novo, classified as likely pathogenic. The patient achieved complete seizure control under levetiracetam monotherapy and continues to show moderate global developmental delay.

This case broadens the genotypic and phenotypic spectrum of SNAP25-related DEE, highlighting febrile illness-triggered episodic ataxia as a previously unreported manifestation. It underscores the relevance of recognizing transient neurological decompensations in the context of SNARE complex dysfunction and highlights the potential for meaningful neurodevelopmental progress despite early epileptic encephalopathy.

## Introduction

Developmental and epileptic encephalopathies (DEE) of infancy and childhood are a group of heterogeneous and treatment-resistant disorders characterized by developmental slowing as a direct result of epileptic activity, the underlying etiology, or a combination of both [1,2]. Over 900 genes and structural causes are linked to DEEs [1].

Soluble N-ethylmaleimide-sensitive factor attachment protein receptor (SNARE) complex, composed of synaptobrevin, syntaxin, and synaptosomal-associated protein 25 (SNAP25), forms the essential fusion machinery for neurotransmitter release, through synaptic vesicle exocytosis [3]. Recent studies have identified mutations in the gene encoding SNAP25 as causative factors for DEEs of infancy and childhood, associated with diverse clinical manifestations [2,3]. To date, 26 pathogenic or likely pathogenic variants have been reported in the Genome Aggregation Database (gnomAD).

The most consistent and well-established clinical features of SNAP25 developmental and epileptic encephalopathy (SNAP25-DEE) include developmental delay, intellectual impairment, and early-onset seizures, typically beginning before two years of age [4]. Additional neurological manifestations include muscular hypotonia, movement disorders (ataxia, dystonia, or tremor), cerebral visual impairment, and brain volume loss, but these are less universally present and show considerable interindividual expressivity [4]. Phenotypic expression is highly heterogeneous, particularly in terms of seizure type, treatment response, severity of neurodevelopmental impairment, and associated cerebellar ataxia or dysmorphic features [2]. The concept of "SNAREopathies" highlights overlapping phenotypes among genes encoding SNARE complex proteins; however, precise genotype-phenotype mapping within SNAP25-related disease remains incompletely characterized [4].

## Case presentation

We report a male child born to non-consanguineous parents. The father had bilateral ptosis without fatigability, ophthalmoparesis, weakness, or ataxia. The mother, three siblings, and one paternal half-brother had no relevant medical history. The maternal family history was notable for three cousins with childhood-onset epilepsy but no impaired neurodevelopment.

Pregnancy was achieved by in vitro fertilization and was uneventful. Delivery occurred at 39 weeks by caesarean section, with Apgar scores of 9/10/10. Birth measurements and the neonatal period were unremarkable.

The child was healthy until seven months of age, when he developed clusters of seizures and status epilepticus in an afebrile context. Seizure semiology included alternating unilateral clonic seizures, generalized tonic-clonic seizures (some beginning with a barking-like vocalization), and brief behavioral arrest without ocular deviation. He eventually developed moderate global developmental delay (without regression), along with a developmental motor coordination disorder. Since the age of two years, febrile illnesses have triggered transient episodes of gait ataxia with no focal weakness, lasting up to one week.

On examination, no dysmorphic features were observed, though mild microcephaly (≈ -2 standard deviation, Nellhaus scale) was noted. Neurological evaluation between episodes of illness revealed no frank gait ataxia, but slight intoeing, foot dragging, and reduced hip elevation during gait. Muscle tone and deep tendon reflexes were normal. The patient was initially treated in his country of origin with phenobarbital (6 mg/kg/day), clonazepam (0,05mg/kg/day), and valproate (~40 mg/kg/day), with incomplete seizure control and mild elevation of transaminases and gamma-glutamyltransferase. At age 32 months, when he first came to our center’s neurology clinic, he was gradually switched to levetiracetam, achieving seizure freedom with high doses.

**Figure 1:**
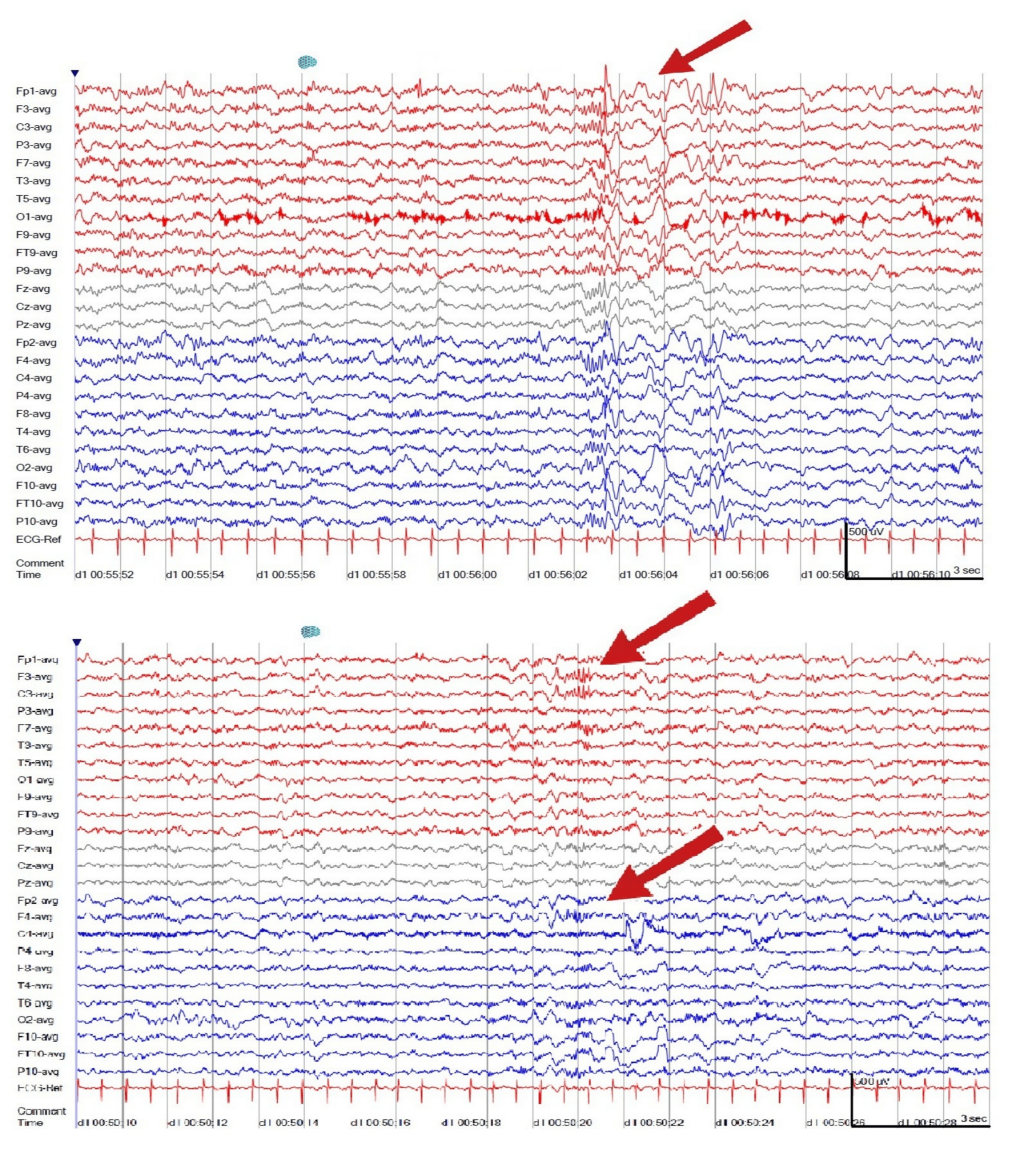
Electroencephalogram showing bilateral frontal epileptiform activity Interictal EEG demonstrates abundant bilateral frontal paroxysmal discharges (arrows), consistent with epileptiform activity. Recording performed at 2 years and 5 months of age.

**Figure 2:**
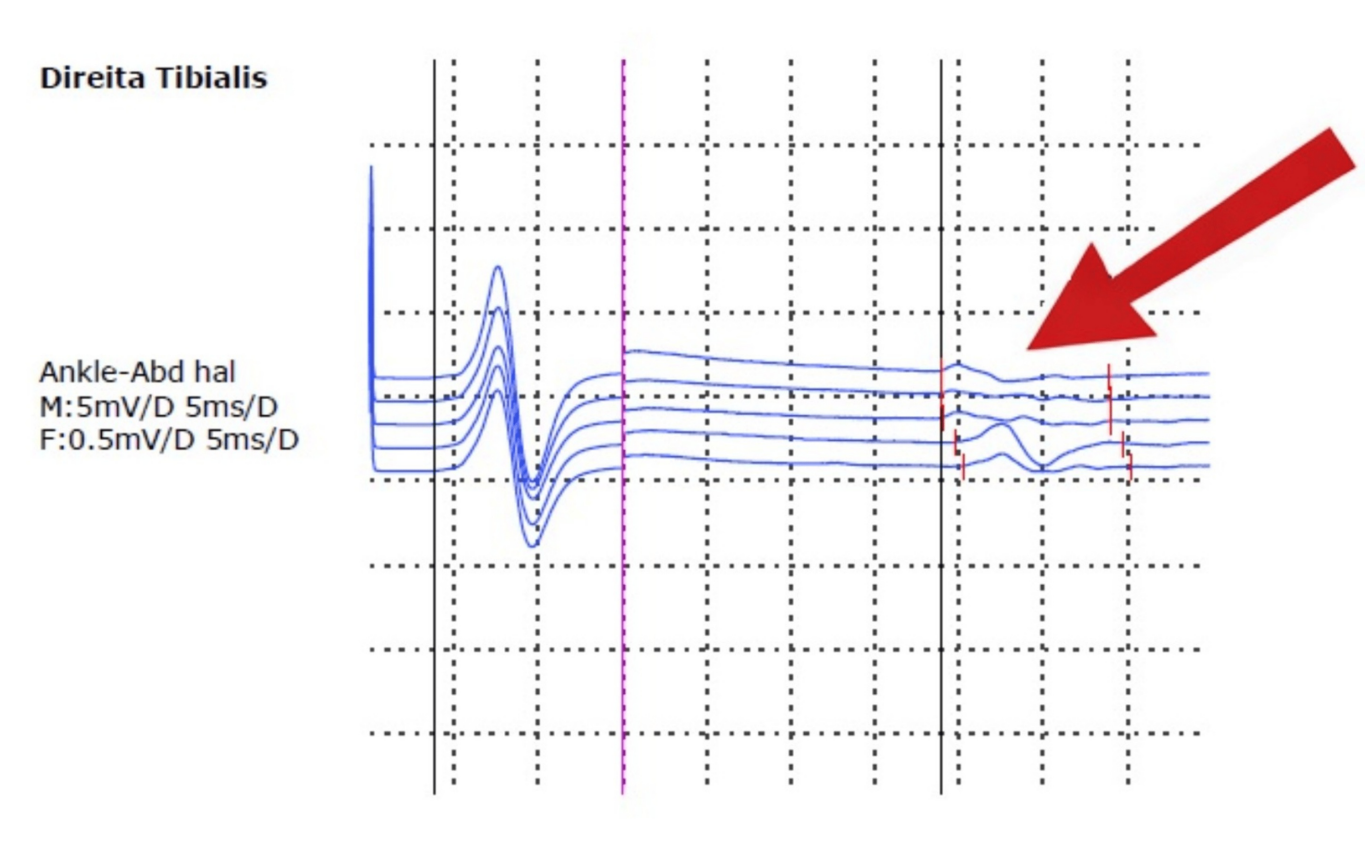
Electromyography of the right tibialis anterior muscle Representative motor unit potentials (arrow) show low-amplitude, short-duration, and simplified morphology, consistent with a myopathic pattern. Nerve conduction velocities and repetitive nerve stimulation were normal (not shown).

Brain magnetic resonance imaging (MRI) was normal. An electroencephalogram (EEG) performed at two years and five months showed abundant bilateral frontal paroxysmal activity (Figure 1). Metabolic investigations were unremarkable, except for transient elevation of 3-hydroxy-isovalerilcarnitine and orotic aciduria, considered valproate-related. Electromyography (EMG) demonstrated myopathic motor unit potentials in the right tibialis anterior, with normal nerve conduction velocities and repetitive nerve stimulation (Figure 2). Given the father’s personal history, he also underwent EMG, which showed borderline myopathic findings in the biceps brachii but no evidence of neuromuscular junction dysfunction. The diagnostic workup is summarized in Table 1.

**Table 1:** Summary of diagnostic workup CDT - carbohydrate-deficient transferrin; CSF - cerebrospinal fluid; EEG - electroencephalogram; EMG - Electromyography; MRI - magnetic resonance imaging;

Investigation	Findings	Interpretation
Brain MRI	Normal	No structural abnormalities identified
EEG	Abundant bilateral frontal paroxysmal activity	Consistent with epileptic encephalopathy
Expanded metabolic panel	Plasma amino acids; CSF amino acids; blood lactate & pyruvate; biotinidase; CDT profile; 24-h urine creatine metabolism: all within normal limits	No evidence of inborn metabolic disorder
Acylcarnitine profile	Transient elevation of 3-hydroxy-isovalerylcarnitine	Considered valproate-related
Urine organic acids	Transient orotic aciduria	Considered valproate-related
Electromyography	Myopathic motor unit potentials in right tibialis anterior; normal nerve conduction velocities; normal repetitive nerve stimulation	No neuromuscular junction dysfunction
Genetic testing	Heterozygous *de novo* splice-site variant in *SNAP25* (NM_003081.5:c.114+2dup), predicted to abolish donor splice site;	Likely pathogenic

A whole-exome-based multigene epilepsy panel identified a heterozygous variant of uncertain significance in SNAP25 (NM_003081.5:c.114+2dup). Subsequent parental analysis demonstrated a de novo origin. The variant affects the canonical +2 donor splice site, and in silico analyses predict disruption of normal splicing. The variant is absent from population databases (gnomAD v4.1), and the patient’s clinical phenotype is highly concordant with SNAP25-DEE. Based on these findings, the variant was reclassified as likely pathogenic according to American College of Medical Genetics and Genomics (ACMG)/ Association for Molecular Pathology (AMP) criteria. Functional validation of aberrant splicing has not yet been performed.

The patient is currently on levetiracetam monotherapy (60 mg/kg/day), with complete seizure control. He has also shown gradual improvement in episodic imbalance and weakness, as well as steady progress in neurodevelopment with ongoing supportive therapies. His expressive language has significantly advanced - he is now able to produce and combine multiple words with good intelligibility. He demonstrates age-appropriate social engagement, naming skills, playfulness, and interaction with peers.

## Discussion

Klöckner et al. [4] described a cohort of 23 individuals with de novo SNAP25 variants, revealing a broad phenotypic spectrum. All presented with developmental delay and varying degrees of intellectual disability and motor impairment. Regression occurred in 29% of cases, some coinciding with seizure onset, which was not observed in our patient. Seizures were reported in 74% of patients, with a median onset at 12 months, encompassing epileptic spasms, generalized and focal seizures, and multiple seizure types over time [4], consistent with our case. Half of the individuals were treated with more than three antiepileptic drugs and still had frequent seizures [4]. Our patient had difficult seizure control initially, but is now on monotherapy with levetiracetam.

Muscular hypotonia, one of the most frequent findings in the cohort, was not described in our patient. It is important to note that we first evaluated him at age 32 months, so earlier hypotonia cannot be ruled out. Although EMG showed myopathic motor unit potentials, he exhibited no weakness, even during febrile illness-related exacerbations. Additional findings included movement disorders such as dystonia, tremor, and ataxia (reported in 33% of cases). None of the patients with ataxia were described as having episodic exacerbations during infections [4]. To our knowledge, there are no prior reports of episodic ataxia or other paroxysmal movement disorders, particularly occurring during febrile illnesses, as seen in our case.

Episodic ataxia is most frequently associated with calcium voltage-gated channel subunit alpha1 A (CACNA1A) defects, particularly episodic ataxia type 2, and is defined by recurrent, transient episodes of cerebellar dysfunction (ataxia, vertigo, dysarthria) that may be triggered by fever, stress, or exertion, with interictal periods of normal or near-normal neurological function [5]. CACNA1A encodes the α1A subunit of the P/Q-type voltage-gated calcium channel, which is essential for presynaptic calcium influx and neurotransmitter release [5]. SNAP25 is a key SNARE protein in the same pathway, modulating calcium channel function and vesicle fusion [2,6]. Functional and structural studies of SNAP25 variants demonstrate disruption of SNARE complex assembly and synaptic transmission, either by impairing protein interactions or destabilizing the complex, leading to defective neurotransmitter release [4]. Although these proteins act at distinct molecular steps, they converge functionally on presynaptic release mechanisms.

Episodic ataxia is most commonly associated with CACNA1A and potassium voltage-gated channel subfamily A member 1 (KCNA1) dysfunction, but may also occur in other genetic epilepsies and metabolic disorders [7]. Proline-rich transmembrane protein 2 (PRRT2) variants are implicated in paroxysmal movement disorders [7,8], particularly paroxysmal kinesigenic dyskinesia, which is characterized by sudden and brief attacks of choreoathetosis or dystonia. Xu et al. [8] described an interaction between PRRT2 and presynaptic proteins, including SNARE components such as SNAP25, suggesting PRRT2 may influence synaptic transmission and contribute to paroxysmal phenotypes through modulation of presynaptic machinery. Severe phenotypes, including episodic ataxia, intellectual disability, and infantile seizures, have been reported in patients with biallelic PRRT2 pathogenic variants [9,10].

Based on this functional convergence, we propose the hypothesis that disruption of synaptic transmission caused by a SNAP25 variant may contribute to the transient, illness-associated ataxia observed in our patient, through mechanisms parallel to those seen in CACNA1A and PRRT2-related disorders, although episodic ataxia is not yet a well-established phenotype of SNAP25-DEE in the literature.

Most individuals with SNAP25-DEE do not present behavioral disturbances [4], in line with our patient’s normal social interaction. Microcephaly has been documented in a minority of cases [3,11] of SNAP25-associated disease.

To our knowledge, this is the first report of recurrent episodic ataxia triggered by febrile illness in SNAP25-associated disease. Strengths of this report include detailed longitudinal characterization, integration of neurophysiological and genetic findings, and the identification of a previously unreported de novo splice-site variant. Limitations include the absence of functional studies to confirm the predicted splicing effect.

## Conclusions

This case highlights the importance of considering SNAP25 variants in infants with early-onset seizures, developmental delay, and episodic neurological decompensations. We report a novel de novo splice-site variant in SNAP25 associated with a clinical phenotype that includes febrile illness-triggered episodic ataxia, representing a candidate phenotypic association that warrants further investigation. Importantly, our findings emphasize the potential for seizure control and neurodevelopmental improvement under tailored therapy, despite persistent neurological vulnerability.
